# Circular RNA circ SET domain containing 2 (circSETD2) inhibits hepatocellular carcinoma cell proliferation and invasion *in vivo* and *in vitro*

**DOI:** 10.1080/21655979.2022.2048577

**Published:** 2022-03-08

**Authors:** Keyan Sun, Lei Zhang, Peng Chen, Debin Qi, Hao Liu, Haili Bao, Xiongwei Wang, Tao Li

**Affiliations:** a Department of General Surgery, Ruijin Hospital, Shanghai Jiao Tong University School of Medicine, Shanghai China; b Department of Neurosurgery, Yangpu Hospital, Tongji University School of Medicine, Shanghai, China

**Keywords:** Circular RNAs, liver cancer, Epithelial-mesenchymal transition

## Abstract

Liver cancer is a common malignant tumor with high incidence and mortality rates. However, a reliable prognostic signature has not yet been confirmed. Circular RNAs (circRNAs) play a role in the development and prognosis of numerous malignancies as well as liver cancer. Therefore, identifying abnormally expressed circRNAs in liver cancer tissue is essential for early diagnosis and treatment. This study found that circular RNA circ SET domain containing 2 (circSETD2) is abnormally expressed in liver cancer tissues, but the role and molecular mechanismsin the occurrence and development of liver cancer are still unclear. The expression level of circSETD2 was evaluated through Quantitative Real-time Polymerase chain reaction (qRT-PCR) in cancerous liver tissues (30 cases), liver cancer cell lines and para-cancerous tissues. Knockdown and overexpression circSETD2 lentiviral vector was constructed and applied to transfect hepatoma cells. Cell Counting Kit-8 (CCK-8), colony formation assay, flow cytometry and Transwell assay were used to examine the effects of circSETD2 overexpression or knockdown on liver cancer migration, invasion, cell cycle and cell proliferation. The tumourigenicity *in vivo* was utilized to assess the effect of circSETD2 on the proliferation of liver cancer cells. circSETD2 expression is lower in cell lines and liver cancer tissues. circSETD2 knockdown can considerably increase liver cancer cells’ invasion, proliferation and colony formation. While *In vitro* and *in vivo*, circSETD2 overexpression shows opposite effect. Western blot showed that circSETD2 knockdown can considerably promote E-cadherin expression and inhibit Vimentin, N-cadherin, matrix metallopeptidase-9 (MMP-9) and MMP-2 expression. These findings improve our understanding of the mechanisms of liver cancer progression and will guide future development of therapeutic strategies against the disease by targeting circ-SETD2.

## GRAPHICAL ABSTRACT

**Figure:**
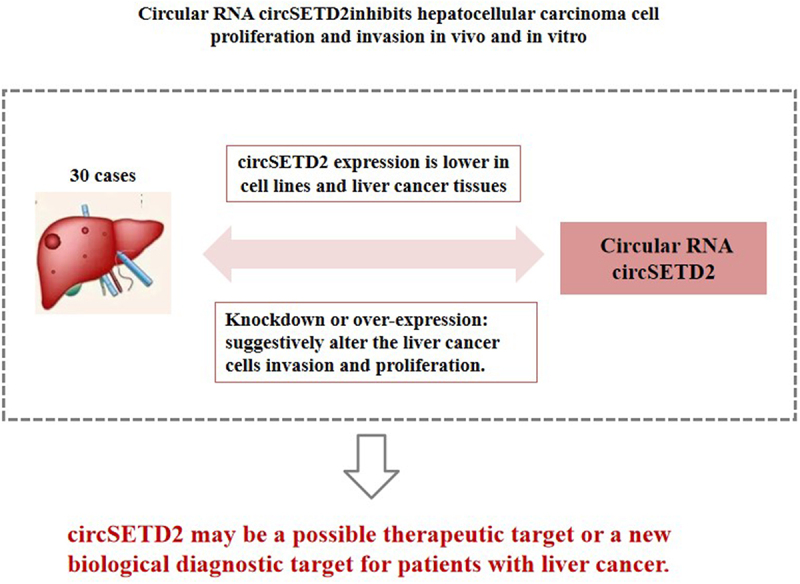

## Introduction

Globally, hepatocellular carcinoma (HCC) is considered one of the most prevalent types of cancer. It is the 3rd most prevalent malignant tumor in terms of mortality after gastric and esophageal cancer [1,2]. Recent research indicates that HCC is a particularly aggressive subtype of serious liver cancer, with a high risk of metastasis and a low survival rate, and accounts for 75%–85% of all liver cancers. As the early symptoms and biomarkers of HCC are difficult to detect, HCC is often detected in its initial stages. Along with the high mortality and metastasis rate, the diagnosis is often made in the late stage, reducing the treatment rate [3,4]. Although current treatment methods, including surgery, chemotherapy, radiotherapy and targeted molecular therapy, continue to improve, the overall 5-year survival rate remains <60% [5]. As a result, the incidence and liver cancer progression mechanisms need to be investigated.

Current research suggests that ncRNA regulatory networks, including miRNA, lncRNA and circular RNA (circRNA), play an essential part in liver cancer incidence and progression [6,7]. circRNA is an endogenous closed RNA that originates from the reverse splicing of pre-RNA. Thus, it lacks 3’ and 5’ structures. circRNAs are common in eukaryotic cells and have gene regulation functions [8,9]. circRNA expression is aberrant in a variety of cancers [10]. The effect of circRNAs on the metabolism of liver cancer cells is one of the current research hotspots [11,12]. For example, circRNA-9119 can prevent liver cancer cell apoptosis by inhibiting the miR-26a/JAK1/STAT3 pathway function. circPIP5K1A can adsorb miR-671-5p by stimulating the PI3K–AKT signaling cascade and boosting gastric cancer growth [13]. circRNAs have stable expression and are not easily degraded by RNase, which leads to a long half-life. They are specifically expressed in a variety of tumors. Therefore, circRNAs have become a novel tumor biomarker for the timely detection and screening of tumors.

circSETD2, also known as has_circ_0065173, is a circular ncRNA (length 4644 bp). It is produced by the reverse splicing of the SETD2 gene located on chromosome 3 [14]. A previous study showed that circSETD2 is downregulated in breast cancer tissues [15]. The overexpression of circSETD2 promotes cell apoptosis whilst inhibiting migration, invasion and cell proliferation. Molecular mechanism studies have indicated that circSETD2 can upregulate signal peptide-CUB-EGF domain-containing protein 2 (SCUBE2) to inhibit breast cancer growth by interacting with miR-155-5p [15]. Furthermore, during the development of the placenta, circSETD2 regulates the proliferation and invasion of trophoblasts through the miR-519a/PTEN signal axis [14]. However, the molecular mechanism and functions of circSETD2 in liver cancer tissues are still unknown. Therefore, this research aims to explore the biological functions and expression of circSETD2 in liver cancer tissues and discover the molecular mechanism of liver cancer to provide possible early diagnosis methods and molecular targets for therapy.

## Materials and methods

### Collection of clinical samples

Thirty individuals with liver cancer who underwent surgery at Ruijin Hospital, Shanghai Jiao Tong University School of Medicine, from April 2019 to December 2020 were recruited. The participants’ age ranged from 38 years to 68 years (average 51.54 ± 10.17 years). There were 16 male and 14 female patients (Table 1). During surgery, cancer and para-cancerous tissues removed from the tumors of patients were collected and placed in a liquid nitrogen tank immediately. The specimens were placed in a − 80°C refrigerator for storage. All specimens were collected from patients who were diagnosed with liver cancer for the first time and underwent surgery. The clinical data and clinicopathological indicators of the enrolled patients were collected. All patients and/or family members who participated signed an informed consensus form. The ethics committee of the hospital examined and approved the present study.

**Table 1.** Clinical baseline data of liver cancer patients

Characteristics	n	%
Age		
>51 years	19	63.33
≤51 years	11	36.67
Gender		
Male	16	53.33
Female	14	46.67
ECOG		
0	9	30.00
1–2	21	70.00
Tumor size		
≤5 cm	11	36.67
>5 cm	19	63.33
BCLC stage		
B	12	40.00
C	18	60.00
Vascular invasion		
Yes	18	60.00
No	12	40.00
Lymph node metastasis		
Yes	13	43.33
No	17	56.67
AFP		
≤400 ng/mL	6	20.00
>400 ng/mL	24	80.00
HBV		
+	16	53.33
-	14	46.67

## Cell culture

The Shanghai Academy of Biological Sciences and the Chinese Academy of Sciences (CAS) Cell Resource Center provided the cell lines needed for this study. The cell lines included human liver cancer cell lines (HB611, SMMC-7721, HepG2, Hep3B and MHCC97L) and human normal liver cells (L02). DMEM (100 U/mL of streptomycin and penicillin and 10% FBS) was used for cell culture. The cells were cultured in a 37°C incubator with 5% CO_2_. According to cell growth characteristics, cell passage was carried out every 2–3 days.

According to the circSETD2 gene sequence, sh-circSETD2 was designed and synthesized by Shanghai Shenggong Biotechnology Company, and a lentiviral vector was constructed. The circSETD2 sequence was amplified and cloned into the pcDNA3.1 vector. The specific steps are mentioned in [14,15]. After counting, hepatic cancerous cells in the exponential phase of development were collected and placed in a 6-well plate, which was then cultured in a 37°C incubator with 5% CO_2_. Before transfection, the cells were washed twice with serum-free culture. Then, a certain amount of virus (MOI) was added, and the cells were incubated for 2 h in a 37°C incubator under gentle shaking every 15 min. After adding 2 mL of culture medium, the culture plates were placed in the incubator for 48 h.

## Quantitative real-time polymerase chain reaction (qRT-PCR)

Total RNA was extracted from cells using Trizol Reagent (Invitrogen, USA)and measure the concentration with Nanodrop2000, and store them at −80°C. According to a previous report, specific primers for related genes were used [15]. GADPH was employed as the reference gene in the reaction, which was carried out in an ABI 7500 PCR system. The circSETD2 and GAPDH primer sequences are as follows [15]: circ_SETD2: (F:5’-CTTGAGAGCTGCCAAAGACCT-3’ and R: 5’-TTGGTGCCTTTGGGCAAAAATCC-3’), GAPDH: (F: 5’-GACTCACTCACGGCAAATTCA-3’ and R: 5’-TCGCTCCTGGAAGATGGTGAT-3’). The specificity of gene amplification was detected by dissolution curve. Each gene’s relative expression was estimated using the 2^−ΔΔCt^ method. The correlation of circSETD2 expression with various clinicopathologic features was determined.

## Cell counting Kit-8 (CCK-8) cell viability

Cell viability was measureed using MTT assay Reagent Kit. Cells were inoculated in a 96-well plate (2000 cells/well) and cultured in a 37°C incubator with 5% CO_2_. Each group should have three replicate wells. The culture duration was set to four time points: 1, 2, 3 and 4 days. About 10 μL of CCK-8 solution was placed in a 96-well plate and placed in a 37°C incubator with 5% CO_2_ for 3 h. After culturing, the sample was placed into a microplate reader, and the wavelength was set to 450 nm to test the absorbance.

## Colony formation assay

After digesting each batch of cells in the exponential phase with 0.25% trypsin, they were pipetted into individual cells. About 700 cells per well were inoculated in a 6-well plate and maintained in a 37°C incubator with 5% CO_2_. After 21 days, the cells were cultured and wash three times with phosphate (PBS) buffer. Add 4% formaldehyde to fix the cell for 10 min and then wash three times with phosphate (PBS) buffer. Add 1 mL of 1% crystal violet to stain for 15 min and wash with phosphate (PBS) buffer for 3 times. Take pictures to observe and count the number of colonies formed.

## Cell cycle analysis

Collect the transfected cells; add 3 mL of PBS to wash cells; add pre-chilled 75% ethanol; fix for 24 h at 4°C; resuspend the cells in 0.5 mL of PI/RNase for staining. Incubate at room temperature for 30 min. Flow cytometry was performed within 1 h after incubation.

## Invasion and migration test by transwell

Matrigel glue planking: Dilute the Matrigel glue at a ratio of 1:4 and then evenly spread it on the bottom of the cell membrane. Transfer it to a 37°C incubator and let it stand for 2 h; ensure Matrigel polymerization is complete. Take each group of cells in the exponential phase. Pipette into single cells after digesting the cell with 0.25% trypsin. Adjust the cell suspension’s cell number to 5 × 10^5^ cells/mL; then add 100 μl of the cell suspension to the Transwell chamber. Then, transfer 600 μL of culture medium (including 20% fetal bovine serum) to a 24-well plate and culture for 36 h at 37°C with 5% CO_2_. Discard the medium after culture. Wipe the cells in the upper section of the chamber with a cotton swab carefully. PBS should be used to clean the bottom surface of the cell membrane, fix it with 95% ethanol for 20 min, and let it air dry. Stain with 1% crystal violet for 20 min. After staining, wash the cell membrane with PBS three times. Observe the air-dried chamber with a microscope at a magnification of 100×, randomly observe the cells in five fields, and take pictures for preservation.

## Western blot

Add protein lysis solution to the cultivated cell line (pre-added protease and phosphatase mixed inhibitor). Then, place it in an ice bath to lyse it and centrifugate at 4°C to collect the supernatant. The supernatant was then quantified using the BCA protein quantification kit. Conventional methods were used to prepare different concentrated gels, and 60–80 μg of samples was added to the gel wells per lane. Electrophoresis should be carried out at a steady pressure of 40 V (concentrated gel)/100 V (separating gel) until the bromophenol blue touches the gel’s edge. For 2 h, seal the culture with skimmed milk powder (8%) at room temperature. Incubate the primary antibody in skimmed milk powder (4%) overnight at 4°C. Then, incubate for 4 h at room temperature with the secondary antibody diluted in skimmed milk powder (4%). TBST should be used to clean the membrane. In the ECL, combine equal parts of liquids A and B. Drop the mixture onto the nitrocellulose membrane in a dropwise fashion and observe through the medical X-ray film.

## Nude mouse tumourigenesis

Eight female BALB/c nude mice (age: 4–6 weeks) were obtained from CAS Shanghai Experimental Animal Center and raised and processed at the SPF-level Experimental Animal Center. Trypsin was applied to digest the cells and then calculated to generate a single-cell suspension. Divide the group into two equal groups, each containing four nude mice. Take 100 μL of 10^6^ live liver cancer cells, mix them with an equal amount of Matrigel, and then inoculate subcutaneously. The tumor volume was measured on the 5th day following inoculation, and mice were sacrificed on the 30th day. The transplanted tumor was stripped for measurement and analysis. The formula V = a*b*b/2 was used to calculate the tumor volume, where a represents the length of the transplanted tumor tissue and b denotes the transplanted tumor tissue width. The transplanted tumor was dissected, and the tumor tissues were stored in liquid nitrogen for measurement and analysis. The total protein and mRNA in the tissue were subsequently extracted for further analysis.

## Statistical analysis

The SPSS16.0 software system was employed for statistical analysis, and all of the experiments were done in triplicate, with the results presented as means ± standard deviation. One-way analysis of variance was utilized to evaluate the differences between the two groups. Student’s t-test was used to compare the two groups. Statistical significance was defined as a p-value less than 0.05.

## Results

### circSETD2 expression is low in liver cancer tissues and cell lines

**Figure 1.**
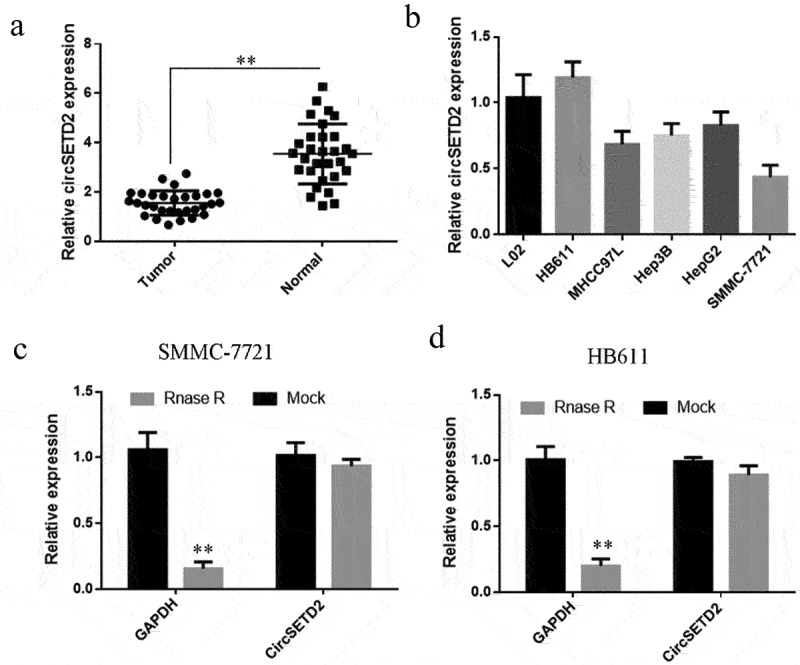
circSETD2 is lowly expressed in liver cancer tissues and cell lines. (a) qRT-PCR was used to detect the expression of circSETD2 in 30 liver cancer tissues and corresponding paracancerous tissues. (b) qRT-PCR was used to detect the expression of circSETD2 in normal liver cells and liver cancer cell lines. (c, d) GAPDH was used as a mock for circSETD2 expression following treatment with 1 mg/mL of RNase-A. **p < 0.01, *p < 0.05.

In various studies, circRNA has been demonstrated to play an essential role in the onset and progression of liver cancer. Therefore, it is urgent to find potential biomarkers for the early diagnosis of liver cancer as well as and new therapeutic targets need to be explored and effective treatment methods. circSETD2 expression was initially detected by qRT-PCR, the results showed that circSETD2 expression was lower in liver cancer tissues compared with paracancerous tissues (Figure 1a). In addition, qRT-PCR was employed to measure the expression of circSETD2 in human normal liver cells (L02) and human liverand liver cancer cell lines (HB611, SMMC-7721, HepG2, Hep3B, and MHCC97L). The findings showed that circSETD2 expression was considerably lower in liver cancer cells compared with human normal liver cells (L02). Amongst them, the expression level of circSETD2 was the lowest in SMMC-7721 cells, whereas the expression difference in HB611 cells was not significant (Figure 1b). As a result, HB611 and SMMC-7721 cells were chosen for further functional testing and molecular mechanism research. Total RNA was isolated from HB611 and SMMC-7721 and processed with Rnase A before reverse transcription to detect the circular properties of circSETD2 further. The qRT-PCR was applied to detect the degradation of circSETD2 by RNase A. The results implied the resistance of circSETD2 to Rnase A digestion, whereas the positive control GAPDH was significantly degraded by RNase A (Figure 1c, d). The above findings suggest that circSETD2 expression is reduced in liver cancer tissues.

## Overexpression or knockdown of circSETD2 can affect liver cancer cell proliferation and cell cycle

**Figure 2.**
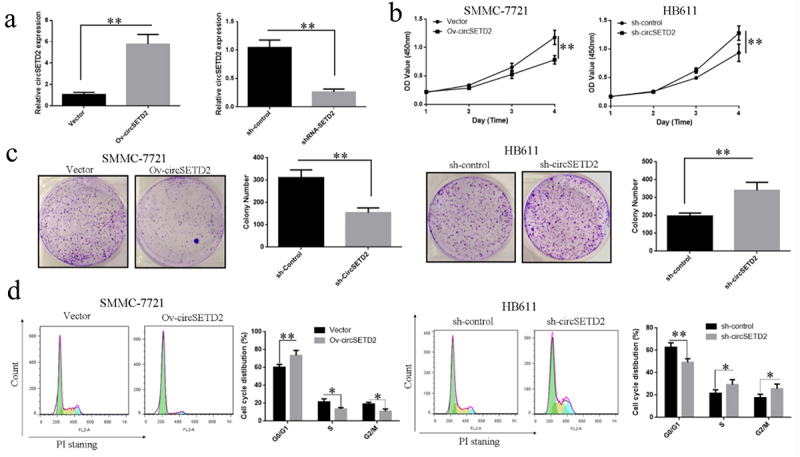
Overexpression of circSETD2 can affect liver cancer cells’ proliferation and cell cycle. (a) qRT-PCR was used to detect the overexpression or knockdown effects of circSETD2 on the expression level of circSETD2 in liver cancer cells (SMMC-7721 and HB611). (b) CCK-8 was used to detect the knockdown effect of circSETD2 on the proliferation of liver cancer cells (SMMC-7721 and HB611). (c) Colony formation experiment was used to measure the effect of circSETD2 knockdown on the proliferation of liver cancer cells (HB611 and SMMC-7721) and colony formation ability. (d) Influence of circSETD2 knockdown on the cycle distribution of liver cancer cells (SMMC-7721 and HB611) identified by flow cytometry. **p < 0.01, *p < 0.05.

To learn more about the effects of circSETD2 on liver cancer cells proliferation, a lentiviral vector targeting circSETD2 sequence (sh-circSETD2) and a blank control vector (sh-Control) were constructed. HB611 cells are transfected, and the circSETD2 level of expression was detected via qRT-PCR. Transfection of the lentiviral vector (sh-circSETD2) targeting the circSETD2 sequence reduced circSETD2 expression levels in liver cancer cells compared with the blank control vector (sh-Control) (Figure 2a). The overexpression vector (Ov-circSETD2) and blank control vector (Vector) of circSETD2 were also constructed. After transfecting SMMC-7721 cells, qRT-PCR detected the expression level of circSETD2 in the cells. The findings revealed that the overexpression vector (Ov-circSETD2) of circSETD2 could significantly upregulate circSETD2 expression levels in liver cancer cells compared with the blank control vector (Vector) (Figure 2a). CCK-8 was used to investigate the effect of circSETD2 knockdown or overexpression on the proliferation of liver cancer cells. The results showed that in SMMC-7721 cells, the overexpression vector of circSETD2 (Ov-circSETD2) could remarkably inhibit the proliferation of liver cancer cells compared with the blank control vector (Vector) (Figure 2b). Moreover, knocking down circSETD2 (sh-circSETD2) could remarkably stimulate HB611 liver cell proliferation compared with the blank control vector (sh-Control) (Figure 2b). Further colony formation assay showed that in SMMC-7721 cells, the overexpression vector of circSETD2 (Ov-circSETD2) could significantly inhibit the cloning ability of liver cancer cells after transfection compared with the blank control vector (Vector) (Figure 2c). Moreover, knocking down circSETD2 (sh-circSETD2) could significantly promote the cloning ability of liver cancer HB611 cells compared with the blank control vector (sh-Control) (Figure 2c). The effect of circSETD2 knockdown or overexpression on the cycle distribution of liver cancer cells was also investigated using flow cytometry. The results showed that in SMMC-7721 cells, the overexpression vector of circSETD2 (Ov-circSETD2) significantly improved the ratio of cells in the G0/G1 phase of the liver cancer cell cycle compared with the blank control vector (Vector).

By contrast, the ratio of cells in the S phase and G2/M phase reduced correspondingly (Figure 2d). After knocking down circSETD2, the blank control vector (sh-Control) (sh-circSETD2) was compared. The proportion of cells in the G0/G1 phase of the liver cancer cell cycle was considerably reduced. By contrast, the proportion of cells in the S and G2/M phases increased (Figure 2d). The abovementioned findings suggest that circSETD2 regulates liver cancer cell growth by influencing the cell cycle.

## circSETD2 overexpression or knockdown affects the capacity of liver cancer cells to invade and migrate

**Figure 3.**
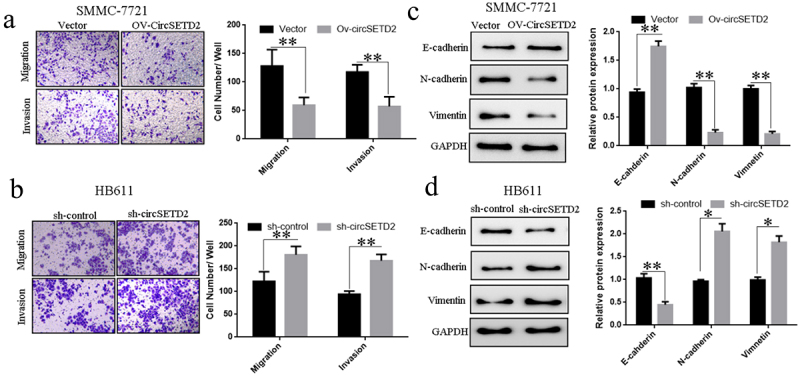
CircSETD2 knockdown or overexpression can impact liver cancer cell (SMMC-7721 and HB611) invasion and migration. (a) Effect of circSETD2 knockdown on the invasion and migration of liver cancer cells (SMMC-772) via Transwell. (b) Effect of Transwell-mediated overexpression of circSETD2 on the invasion and migration of liver cancer cells (HB611). (c) Western blot was utilized to assess the effect of circSETD2 silencing on the expression of proteins involved in the invasion and migration of liver cancer cells (SMMC-7721). (d) Effects of circSETD2 overexpression on the expression of HCC cell (HB611) invasion and migration–related proteins identified through Western blot. *p < 0.05, **p < 0.01.

The earlier findings suggest that overexpression or knockdown of CircSETD2 circSETD2 significantly impacts liver cancer cells’ cells’ proliferation and cell cycle.Transwell results showed that in SMMC-7721 cells, the invasion and migration ability of liver cancer cells was significantly reduced after transfection of circSETD2 overexpression vector (Ov-circSETD2) compared with the blank control vector (Figure 3a). The capacity of liver cancer HB611 cells to invade and migrate was dramatically promoted after knocking down circSETD2 (sh-circSETD2) compared with the blank control vector (sh-Control) (Figure 3b). The influence of circSETD2 on the expression of HCC cell invasion and migration–related proteins was further detected by Western blot. The results demonstrated that, compared with the blank control vector (Vector), the overexpression model might dramatically promote E-cadherin expression in liver cancer cells whilst decreasing N-cadherin and Vimentin expression in SMMC-7721 cells (Figure 3c). Compared with the blank control vector (sh-Control), the knockdown model could significantly suppress E-cadherin expression in liver cancer HB611 cells whilst promoting N-cadherin Vimentin expression (Figure 3d). The above results indicate that circSETD2 affects the invasion and migration of liver cancer cells by regulating the expression of E-cadherin, N-cadherin and Vimentin.

## circSETD2 overexpression prevents the progression of liver cancer cells *in vivo*

**Figure 4.**
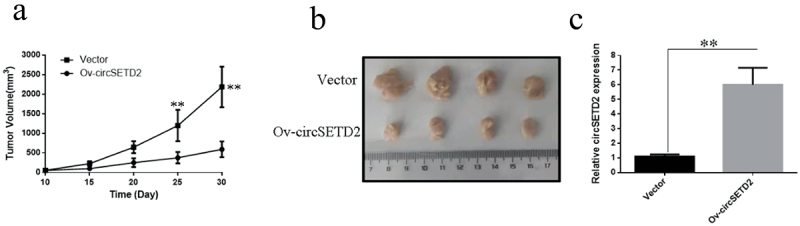
circSETD2 overexpression in nude mice dramatically inhibits the growth of liver cancer cells (SMMC-7721). (a) Effect of circSETD2 overexpression on the growth of liver cancer cells in nude mice. (b) Subcutaneous transplanted tumor tissue was stripped 30 days after the transplanted tumor. (c) qRT-PCR detection of circSETD2 expression in transplanted tumor tissues. **p < 0.01, *p < 0.05.

To further validate the effect of CircSETD2 on liver cancer cells proliferation *in vivo*, SMMC-7721 cells were inoculated subcutaneously in nude mice, and the size (including length and width) of the transplanted tumor was recorded on the 10th day after inoculation and measured every 5 days. The measurements were used to create a growth curve for the transplanted tumor. The results reveal that the overexpression vector (Ov-circSETD2) significantly inhibits liver cell proliferation in nude mice compared with the blank control vector (Vector) (Figure 4a). On the 30th day, nude mice were sacrificed, and the transplanted tumor tissue was stripped and photographed. The results indicate that the overexpression of circSETD2 (Ov-circSETD2) effectively inhibits liver cancer cell proliferation *in vivo* (Figure 4b). qRT-PCR showed that circSETD2 was present in transplanted tumor tissues. circSETD2 was more likely to be found in transplanted tumor tissue from patients who had overexpressed circSETD2 (Ov-circSETD2) than those who had a blank control vector (No-circSETD2) (Vector). The above experiments show that when circSETD2 is overexpressed in nude mice, it can significantly decrease the proliferation of liver cancer cells.

## Discussion

Primary liver cancer is a malignant tumor with a high fatality rate, accounting for about 90% of liver cancer. Most patients with liver cancer usually have a poor prognosis, often accompanied by the risk of metastasis and recurrence [16]. Thus, the search for promising biomarkers for early detection of liver cancer and novel therapeutic targets and successful treatment approaches is critical. circRNAs are closed circular molecules expressed at specific tissues and developmental stage levels [13]. Recent studies have proved that circRNA dysregulation plays an essential regulatory role in the occurrence and development of cancer [17]. For instance, the expression of cSMARCA5 in liver cancer tissues is low, and the downregulation of cSMARCA5 is linked with clinicopathological features of liver cancer patients (such as aggressiveness); after hepatectomy, it is an independent risk factor for overall survival and recurrence-free survival. cSMARCA5 suppresses liver cancer cell proliferation and migration in both *in vivo* and *in vitro* studies [18]. circMET expression is increased in liver cancer tissues, and its level is associated with patient survival and recurrence. The overexpression of circMET promotes the development of liver cancer by initiating epithelial–mesenchymal transition (EMT) and strengthening the immunosuppressive tumor microenvironment [19]. However, the functions of most circRNAs in HCC remain uncertain. circSETD2 expression was shown to be reduced in liver cancer tissues and cell lines for the first time in this investigation. circSETD2 overexpression prevents the proliferation, colony formation, invasion and migration of liver cancer cells *in vitro*, and vice versa. Furthermore, *in vivo* nude mouse tumourigenic tests determined that circSETD2 overexpression can significantly prevent liver cancer cell proliferation.

In recent years, the identification and clarification of the expression and role of circRNAs in different tumor tissues have become the latest research hotspot in the RNA field [7,9]. According to a growing number of studies, many circRNAs have cell-specific expression and are linked to physiological development and different disorders, especially in tumor tissues [20]. For instance, the high expression of hsa_circ_0003141 in liver cancer tissues is related to poor survival rate in patients with liver cancer. Reduced expression of hsa_circ_0003141 induces apoptosis in cells and inhibits proliferation and invasion. Thus, hsa_circ_0003141 behaves as an oncogene [21]. In comparison, circ-0001649 is lowly expressed in HCC cell lines and tumor tissues. The overexpression of circ-0001649 has been found *in vivo* and *in vitro* to suppress liver cancer cell proliferation and migration significantly. As a result, circ-0001649 functions as a tumor suppressor gene [22]. Additionally, research has demonstrated that circRNAs can be a valuable biomarker for liver cancer. For instance, compared with normal samples, hsa_circ_0004001, hsa_circ_0075792 and hsa_circ_0004123 are substantially expressed in blood samples of liver cancer patients; furthermore, ROC analysis demonstrates that the combination of these three circRNAs is an excellent diagnostic biomarker in HCC [23].

Thus, discovering important circRNAs implicated in the development and onset of HCC and elucidating their specific mode of action would provide novel ideas for detecting and treating HCC. This is the first study demonstrating that circSETD2 is significantly downregulated in liver cancer tissues and cell lines. CCK-8 and colony formation assays demonstrated that transfecting SMMC-7721 cells with a circSETD2 overexpression plasmid dramatically prevents colony formation and cell proliferation and induces cell cycle arrest. circSETD2 silencing can greatly enhance HB611 cell proliferation, colony formation and cell cycle advancement. The recurrence and metastasis of liver cancer are essential factors affecting long-term survival of patients. More than 70% of liver cancer patients after radical clinical, surgical resection or liver transplantation can have tumor recurrence or distant metastasis. More than 90% of death factors are related to tumor metastasis and recurrence [24]. EMT is the most important way for tumor cells to obtain invasion and metastasis during the initial stage of liver cancer metastasis. This process is mainly designed to reduce the expression of adhesion molecule E-cadherin, whereas the expression of N-cadherin and Vimentin proteins increases [25]. Thus, preventing liver cancer invasion and metastasis is the primary research focus and therapeutic method for cancer. Additionally, this study established that circSETD2 overexpression strongly inhibits the invasion and migratory capacity of SMMC-7721 cells, promotes E-cadherin expression and inhibits N-cadherin and Vimentin expression. Therefore, circSETD2 may act as a tumor suppressor gene in liver cancer tissues. Furthermore, additional *in vivo* investigations have demonstrated that circSETD2 overexpression inhibits liver cancer cell proliferation significantly. However, the mechanism by which circRNA influences tumor cell biological behavior is complicated. The mechanism by which circSETD2 affects the biological behavior of liver cancer cells needs to be investigated further.

## Conclusion

This study demonstrated that circSETD2 expression is low in cell lines and liver cancer tissues. *In vitro*, circSETD2 overexpression strongly inhibits the proliferation, colony formation, invasion and migration of liver cancer cells, whereas circSETD2 knockdown exerts the opposite effects. Furthermore, *in vivo*, nude mouse tumourigenic tests determined that circSETD2 overexpression significantly inhibits the proliferation of liver cancer cells. Finally, this study demonstrates that circSETD2 functions as a tumor suppressor gene in HCC. In the future, circSETD2 could be a new target gene for liver cancer therapy.
